# Supplementary material for: Next Generation of Ovarian Cancer Detection Using Aptamers
Source: Int J Mol Sci. 2023 Mar 28;24(7):6315. doi: 10.3390/ijms24076315 (PMC10094455; doi:10.3390/ijms24076315)
Supplement: Supplementary file 1 [file ijms-24-06315-s001.zip › Table S1 and S2.pdf]

Table S1. Relation of membrane proteins selected as potential targets for aptamers in Caov-3 cells.

Protein	Gene	Uniprot ID	Uniprot Cell Location	PSORT Cell Location	Transmembrane Domain	Topology Membrane*
Secreted and transmembrane protein 1	SECTM1	Q8WVN6	Plasma membrane	Endoplasmic reticulum	1	1a
Integrin beta-4	ITGB4	P16144	Plasma membrane	Extracellular region	1	1a
MHC class I polypeptide-related sequence B	MICB	Q29980	Plasma membrane	Endoplasmic reticulum	1	1a
Oxidized low-density lipoprotein receptor 1	OLR1	P78380	Plasma membrane	Nucleus	1	2
Intercellular adhesion molecule 2	ICAM2	P13598	Plasma membrane	Golgi Complex	1	1a
Receptor-transporting protein 4	RTP4	Q96DX8	Plasma membrane	Cytoplasm	1	Nt
HLA class I histocompatibility antigen, alpha chain F	HLA-F	P30511	Plasma membrane	Endoplasmic reticulum	1	1a
Integrin beta-2	ITGB2	P05107	Plasma membrane	Endoplasmic reticulum	1	1a
Low-density lipoprotein receptor-related protein 8	LRP8	Q14114	Plasma membrane	Extracellular region	1	1a

**FXYD domain-containing ion transport regulator 3**	**FXYD3**	**Q14802**	**Plasma membrane**	**Endoplasmic reticulum**	**1**	**1a**
**Protein NDRG4**	**NDRG4**	**Q9ULP0**	**Plasma membrane**	**Endoplasmic reticulum**	**1**	**2**
**Inactive Rhomboid protein 2**	**RHBDF2**	**Q6PJF5**	**Plasma membrane**	**Plasma membrane**	**7**	**3b**
**Filamin-A**	**FLNA**	**P21333**	**Plasma membrane**	**Endoplasmic reticulum**	**1**	**2**
**Alkaline phosphatase, placental type**	**ALPP**	**P05187**	**Plasma membrane**	**Plasma membrane**	**1**	**1a**
**Cadherin-5**	**CDH5**	**P33151**	**Plasma membrane**	**Endoplasmic reticulum**	**1**	**1a**
**Acid sphingomyelinase-like phosphodiesterase 3b**	**SMPDL3B**	**Q92485**	**Plasma membrane**	**Plasma membrane**	**1**	**1a**
**Sushi domain-containing protein 2**	**SUSD2**	**Q9UGT4**	**Plasma membrane**	**Plasma membrane**	**1**	**1a**
**Metalloreductase STEAP4**	**STEAP4**	**Q687X5**	**Plasma membrane**	**Endoplasmic reticulum**	**5**	**3a**
**Neuronal cell adhesion molecule**	**NRCAM**	**Q92823**	**Plasma membrane**	**Cytoplasm**	**1**	**Nt**
**Suppressor of tumorigenicity 14 protein**	**ST14**	**Q9Y5Y6**	**Plasma membrane**	**Mitochondria**	**1**	**2**

**Claudin-7**	**CLDN7**	**O95471**	**Plasma membrane**	**Endoplasmic reticulum**	**3**	**3a**
**Ferric-chelate reductase 1**	**FRRS1**	**Q6ZNA5**	**Plasma membrane**	**Plasma membrane**	**6**	**3b**
**Adhesion G-protein coupled receptor G1**	**GPR56**	**Q9Y653**	**Plasma membrane**	**Plasma membrane**	**8**	**3b**
**Excitatory amino acid transporter 1**	**SLC1A3**	**P43003**	**Plasma membrane**	**Plasma membrane**	**9**	**3b**
**Immunoglobulin superfamily member 3**	**IGSF3**	**O75054**	**Plasma membrane**	**Plasma membrane**	**1**	**1a**
**Mesothelin**	**MSLN**	**Q13421**	**Plasma membrane**	**Plasma membrane**	**1**	**1a**

* Membrane topology: (1a) a cleavable signal sequence and one transmembrane segment, (2) N-terminal portion inserted in the cytoplasmic region, (Nt) uncleavable signal peptide and one transmembrane segment near its C-terminus, (3a) multiple transmembrane regions with its N-terminus facing the cytosolic side, (3b) multiple transmembrane regions with its N-terminus facing the cytosolic side.

Table S2. Relation of membrane proteins selected as potential targets for aptamers in Ovcar-3 cells.

**Protein**	**Gene**	**Uniprot ID**	**Uniprot Cell Location**	**PSORT Cell Location**	**Transmembrane Domain**	**Topology Membrane***
**Claudin-6**	CLDN6	P56747	Plasma membrane	Endoplasmic reticulum	4	3a
**Transmembrane protein 100**	TMEM100	Q9NV29	Plasma membrane	Endoplasmic reticulum	3	3a
**Very low-density lipoprotein receptor**	VLDLR	P98155	Plasma membrane	Extracellular region	1	1a

**Sphingomyelin phosphodiesterase 2**	SMPD2	O60906	Plasma membrane	Endoplasmic reticulum	1	2
**Heat shock protein HSP 90-beta**	HSP90AB1	P08238	Plasma membrane	Cytoplasm	1	Nt
**Matrix metalloproteinase-15**	MMP15	P51511	Plasma membrane	Endoplasmic reticulum	1	1a
**Atrial natriuretic peptide receptor 3**	NPR3	P17342	Plasma membrane	Golgi Complex	1	1a
**HLA class I histocompatibility antigen, A alpha chain**	HLA-A	P004439	Plasma membrane	Endoplasmic reticulum	1	1a
**MANSC domain-containing protein 1**	MANSC1	Q9H8J5	Plasma membrane	Cytoplasm	1	Nt
**Protein jagged-1**	JAG1	P78504	Plasma membrane	Endoplasmic reticulum	2	3a
**Killer cell lectin-like receptor subfamily G member 2**	KLRG2	A4D1S0	Plasma membrane	Nucleus	1	2
**Uroplakin-1b**	UPK1B	O75841	Plasma membrane	Plasma membrane	4	3a
**Ephrin type-A receptor 1**	EPHA1	P21709	Plasma membrane	Endoplasmic reticulum	1	1a
**Neuronal cell adhesion molecule**	NRCAM	Q92823	Plasma membrane	Cytoplasm	1	Nt
**Suppressor of tumorigenicity 14 protein**	ST14	Q9Y5Y6	Plasma membrane	Mitochondria	1	2

**Transmembrane channel-like protein 4**	TMC4	Q7Z404	Plasma membrane	Plasma membrane	8	3a
**Claudin-7**	CLDN7	O95471	Plasma membrane	Endoplasmic reticulum	3	3a
**Transmembrane protein 100**	TMEM100	Q9NV29	Plasma membrane	Endoplasmic reticulum	3	3a
**Tetraspanin-15**	TSPAN15	O95858	Plasma membrane	Endoplasmic reticulum	4	3a
**Adhesion G-protein coupled receptor G1**	GPR56	Q9Y653	Plasma membrane	Plasma membrane	8	3b
**Immunoglobulin superfamily member 3**	IGSF3	O75054	Plasma membrane	Plasma membrane	1	1a
**Uroplakin-3b**	UPK3B	Q9BT76	Plasma membrane	Extracellular region	0	-
**Leukocyte surface antigen CD47**	CD47	Q08722	Plasma membrane	Endoplasmic reticulum	5	3a
**Mesothelin**	MSLN	Q13421	Plasma membrane	Plasma membrane	1	1a

* Membrane topology: (1a) a cleavable signal sequence and one transmembrane segment, (2) N-terminal portion inserted in the cytoplasmic region, (Nt) uncleavable signal peptide and one transmembrane segment near its C-terminus, (3a) multiple transmembrane regions with its N-terminus facing the cytosolic side.
