# Supplementary figures and images for: Next Generation of Ovarian Cancer Detection Using Aptamers
Source: Int J Mol Sci. 2023 Mar 28;24(7):6315. doi: 10.3390/ijms24076315 (PMC10094455; doi:10.3390/ijms24076315)

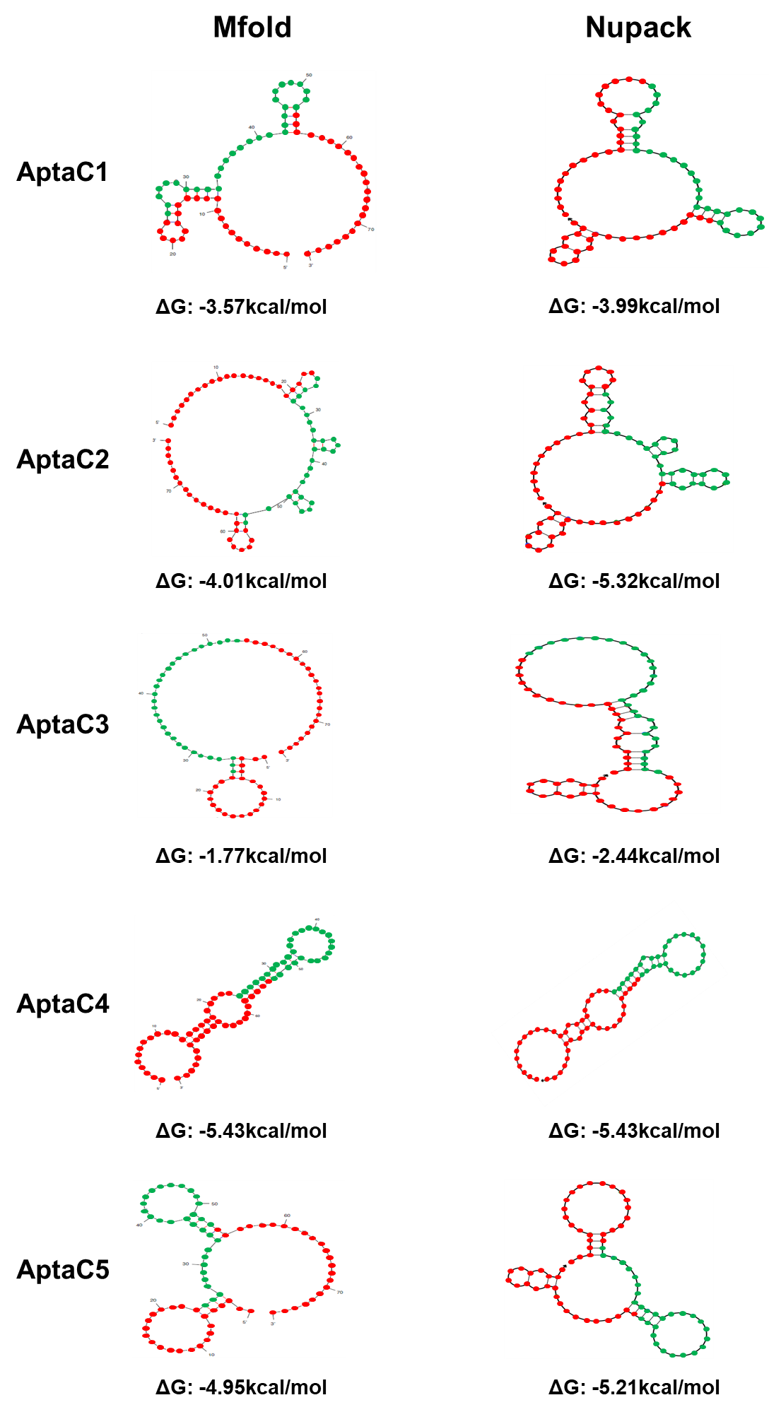

Supplement: Supplementary file 1 [file ijms-24-06315-s001.zip › Figure S1.tif]

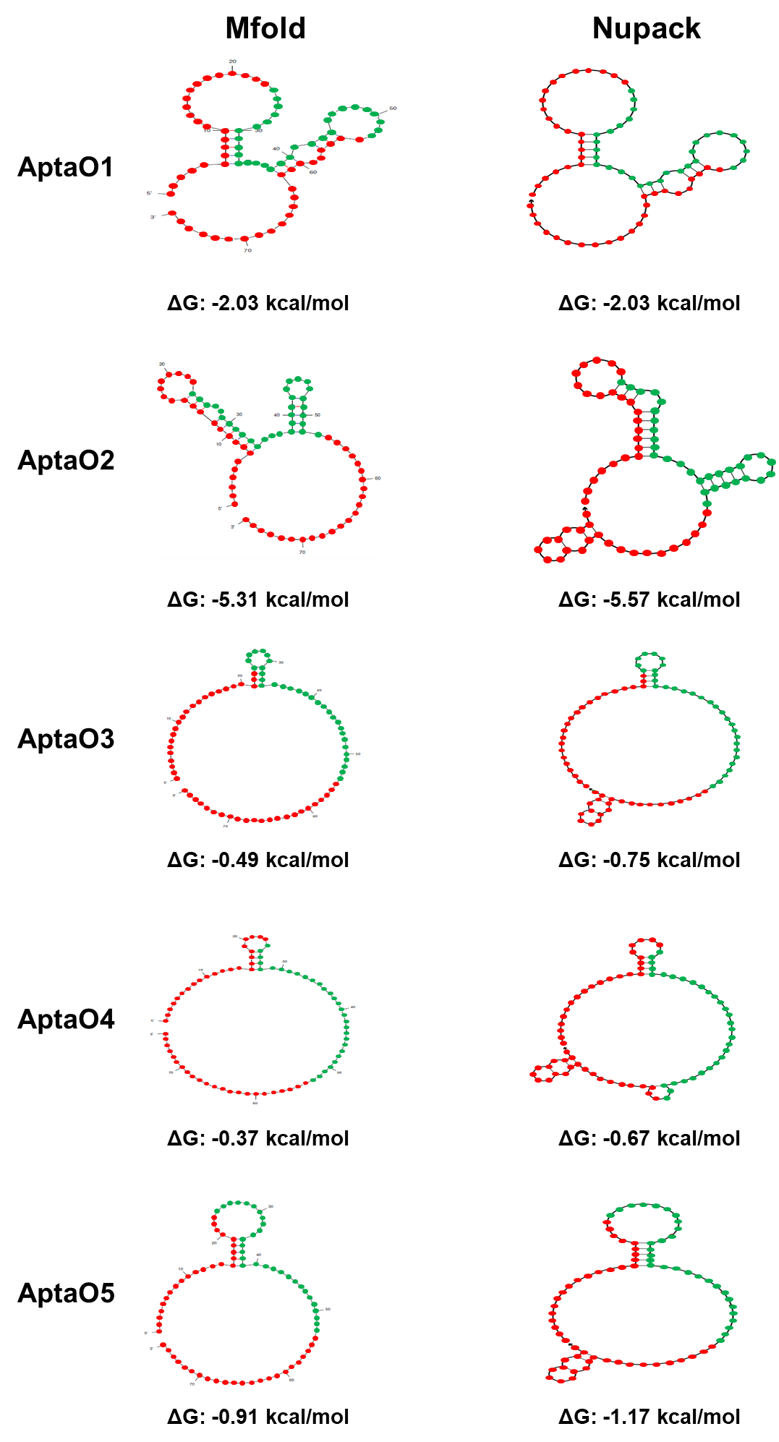

Supplement: Supplementary file 1 [file ijms-24-06315-s001.zip › Figure S2.tif]

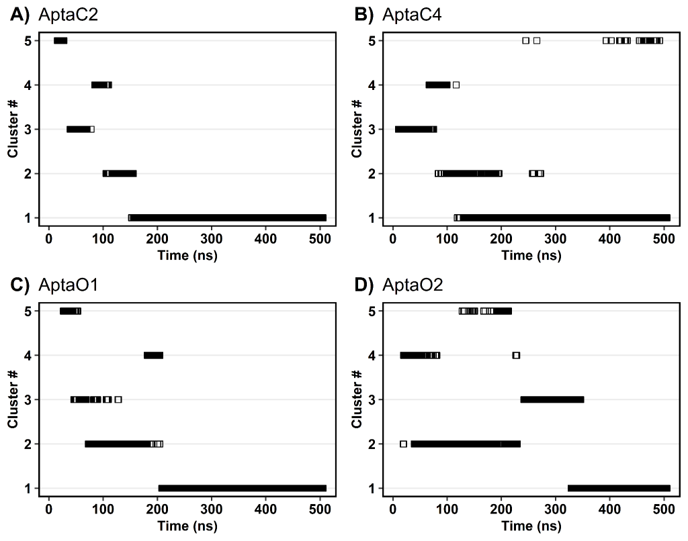

Supplement: Supplementary file 1 [file ijms-24-06315-s001.zip › Figure S3.tif]
